# Ovarian Carcinoma Presenting With a Large Cervical Mass

**DOI:** 10.7759/cureus.20994

**Published:** 2022-01-06

**Authors:** Melek Tugce Yilmaz, Ezgi Gurlek, Melis Gultekin, Korhan Kahraman, Mehmet C Salman, Alp Usubutun, Deniz Akata, Eser Lay Ergun, Zafer Arik, Ferah Yildiz

**Affiliations:** 1 Department of Radiation Oncology, Hacettepe University Faculty of Medicine, Ankara, TUR; 2 Department of Obstetrics and Gynecology, Bahçeşehir University Faculty of Medicine, Istanbul, TUR; 3 Department of Obstetrics and Gynecology, Hacettepe University Faculty of Medicine, Ankara, TUR; 4 Department of Pathology, Hacettepe University Faculty of Medicine, Ankara, TUR; 5 Department of Radiology, Hacettepe University Faculty of Medicine, Ankara, TUR; 6 Department of Nuclear Medicine, Hacettepe University Faculty of Medicine, Ankara, TUR; 7 Department of Medical Oncology, Hacettepe University Faculty of Medicine, Ankara, TUR

**Keywords:** radiology, cervical mass, ovarian carcinoma, gynecologic cancers, uterine cervical cancer

## Abstract

Cervical metastasis in ovarian cancer is a rare entity. Therefore, care should be taken in the differential diagnosis of cervical masses as it may mimic a primary tumor. This report aimed to emphasize the importance of a multidisciplinary approach in these tumors. We present a case of a 73-year-old female who presented with post-menopausal vaginal bleeding and cervical mass. The patient was diagnosed with ovarian carcinoma with a multidisciplinary approach. Although cervical metastasis of ovarian cancer is rare, the possibility of secondary cancer should be kept in mind, especially in cervical tumors with atypical clinical course.

## Introduction

Ovarian cancer is the seventh most common cancer among women worldwide [1]. In 2020, 313,959 new ovarian cancers were diagnosed worldwide, and 207,252 deaths due to ovarian cancer occurred [2].

Most ovarian cancer patients present at an advanced stage disease (stage III-IV) [3]. Ovarian cancer mainly metastasizes via intraperitoneal and lymphatic pathways and rarely develops blood-borne metastases. The most common metastatic sites are the peritoneum, liver, and lymph nodes [4] and rarely the bones and brain [5].

Metastasis of ovarian cancer to the cervix is ​​rarely seen and presented as case reports in the literature [6, 7]. Herein, we present a case of ovarian cancer with cervical metastasis at the time of diagnosis.

## Case presentation

A 73-year-old G5P5 Caucasian female patient with a chief complaint of post-menopausal vaginal bleeding was referred from another health care center with a locally advanced cervical cancer diagnosis. Abdomen computed tomography was performed outside our hospital. There were masses in both adnexa and cervix, and the pathological evaluation of cervical biopsy was reported as squamous cell carcinoma (SCC). 

**Figure 1:**
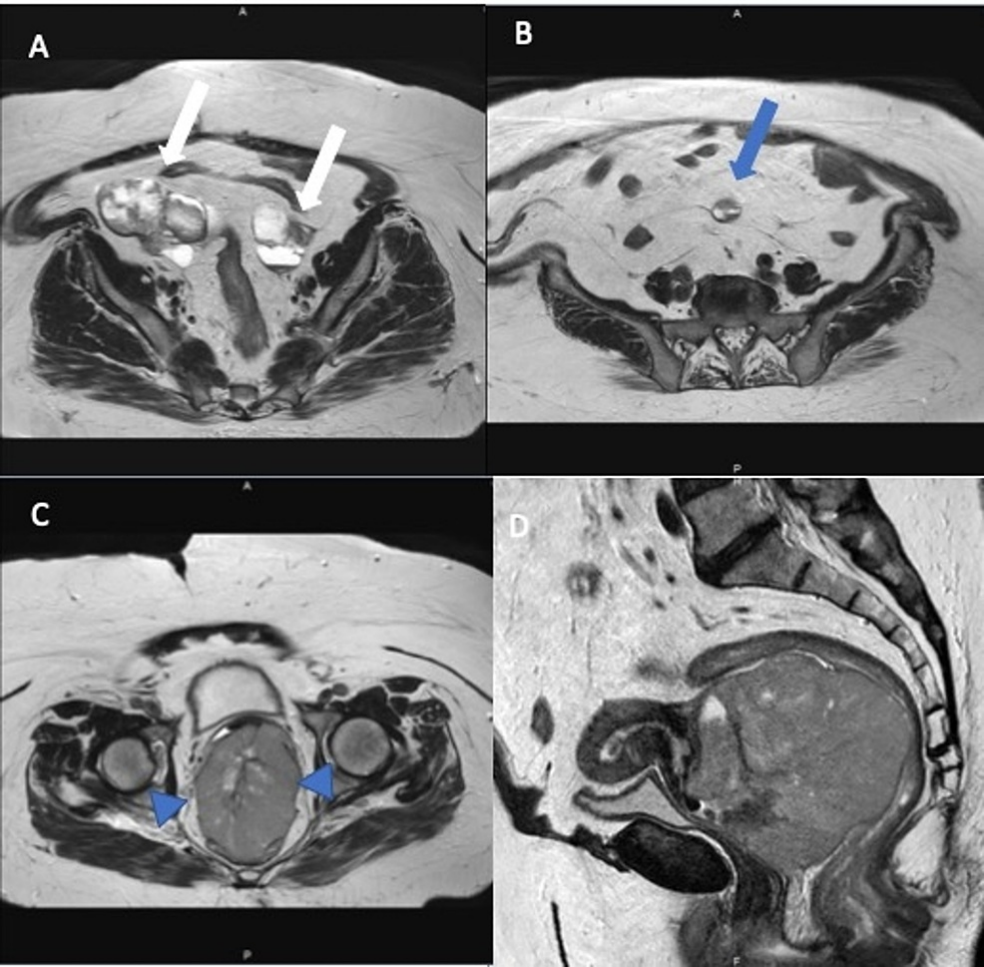
Pelvic MRI images. A: Axial T2-weighted image shows multicystic neoplastic masses (white arrows) with solid components in both ovaries. B: A peritoneal implant (blue arrow) with similar characteristics to the ovarian lesions is seen in the peritoneal cavity. C and D: T2-weighted images of the uterine cervix in the axial and sagittal plane reveal the bulky cervical tumor. The cervical stromal ring is well preserved (arrowhead). However, the anterior rectal wall continuum is not very well seen.

On physical examination, the abdomen was soft, and no organomegaly or lymphadenopathy was detected. On per speculum examination, a large necrotic, highly vascular, fragile mass occupying the whole cervix and extending to the upper third of the vagina was observed. Bimanual examination revealed no rectal invasion, and bilateral parametrium was not infiltrated. Laboratory tests revealed high serum cancer antigen 125 (CA 125) levels (313 U/mL). In the patient's pelvic MRI, multilocular multicystic neoplastic masses with solid components were observed measuring 8x5 cm and 5x4 cm in size in the right and left ovary, respectively (Figure 1).

**Figure 2:**
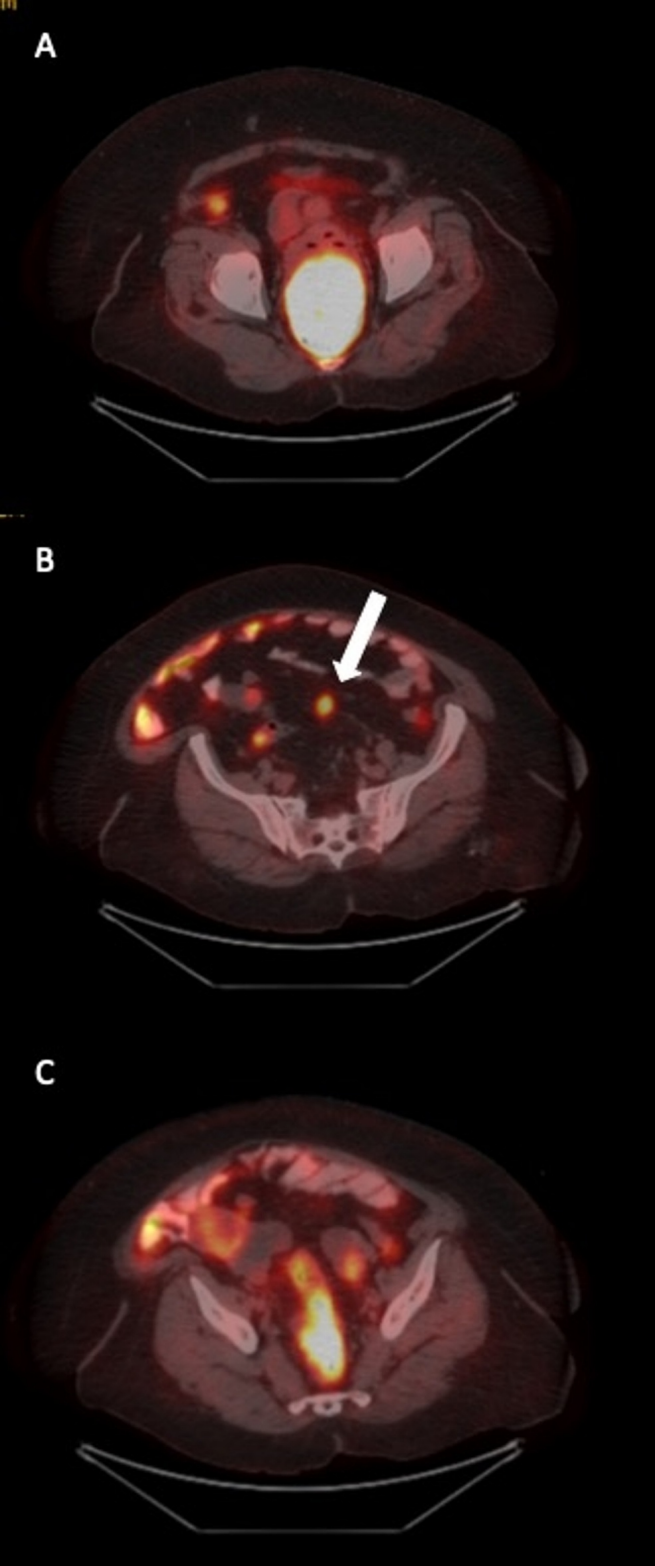
18 Fluoro-2-deoxyglucose positron emission tomography images. A: Axial image, increased FDG uptake in the mass measuring 9 x 7 x 9 cm in the cervix. B: Axial image, peritoneal implant. C: Axial image, increased FDG uptake in the ovarian masses. FDG: 18 Fluoro-2-deoxyglucose.

In addition, there was a well-circumscribed 9.5 x 7.4 cm mass with contrast enhancement and diffusion restriction, with areas of necrosis in the cervix. The huge mass in the cervix mostly filled the vagina and exerted significant pressure on the rectum wall. In sagittal images, it was seen that the integrity of the rectum wall was disrupted in some areas. However, the cervical stromal ring was intact in T2- weighted images. A tumor implant with a diameter of 2 cm, similar to the masses in the ovary, was observed in the sigmoid mesocolon. The 18 fluoro-2-deoxyglucose positron emission tomography (FDG-PET) demonstrated increased FDG uptake with a maximum standardized uptake value (SUVmax) of 24 in the cervical mass measuring 9x7x9 cm and pathological FDG uptake was observed in the implant located in the pelvic peritoneum with a SUVmax value of 4.2. In addition, there was increased FDG uptake with a SUVmax value of 4 in nodular soft tissue masses in the bilateral adnexa (Figure 2).

The patient's previous biopsy specimen was re-evaluated and reported as undifferentiated carcinoma. No further interpretation could be made due to the small amount of the tumor with an immunohistochemical study showing p16 positivity and p63 and p40 negativity. The patient was discussed on the gynecological oncology tumor board. Due to MRI findings of the patient, which were not compatible with primary cervical cancer, a re-biopsy was recommended. Since the patient had severe bleeding, it was decided to apply palliative radiotherapy (RT) until the pathology result was clarified. The patient received 30 Gy in 10 fractions. The treatment was well-tolerated, and the patient's bleeding stopped on the third day of treatment.

**Figure 3:**
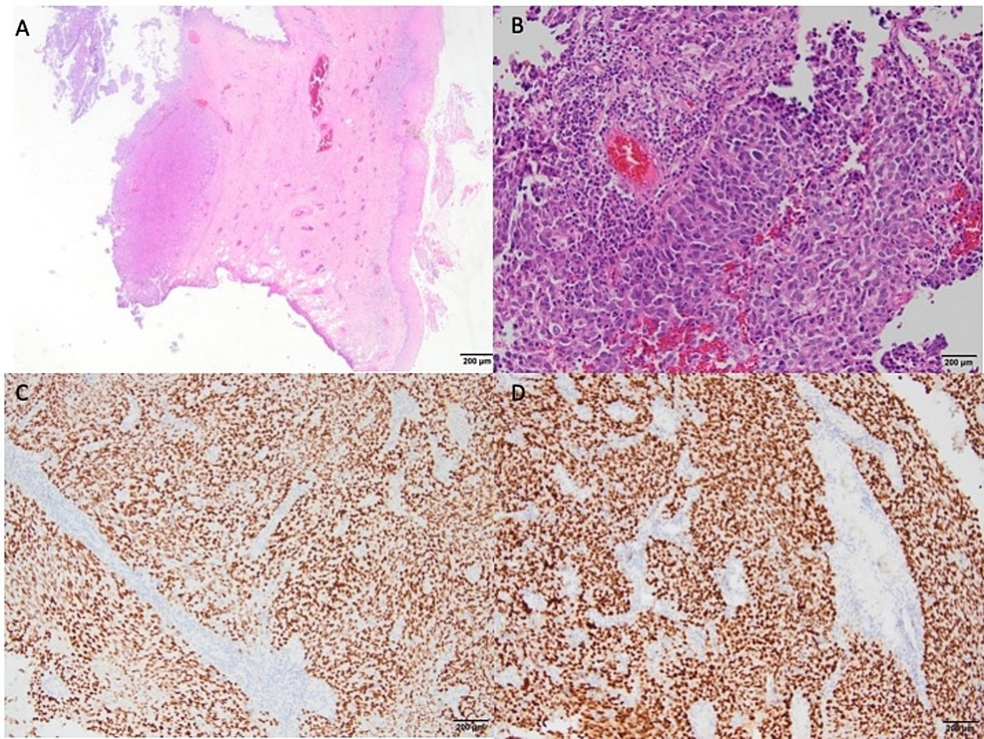
A: A tumor nodule is seen under the squamous epithelium in the cervical biopsy specimen (H&E) (x40). B: A high-power view of the solid tumor with atypical cellular features is seen (H&E) (x200). C: Immunohistochemically, the tumor cells show diffuse nuclear staining with WT-1 (x100). D: Immunohistochemically, the tumor cells show diffuse nuclear staining with PAX-8 (x100).

The second biopsy from the cervical mass was reported as high-grade tubo-ovarian serous carcinoma (Figure 3).

The tumor showed solid morphology and immunohistochemical staining with CK7, WT-1, Pax8, and ER. Also, there was mutant-type staining of p53. PR and p40 were reported to be negative. As a result of immunohistochemical staining and accompanied radiological findings, the patient was diagnosed with high-grade serous tubo-ovarian cancer. The patient was staged as stage IIIC ovarian cancer according to the 2014 International Federation of Gynaecology and Obstetrics (FIGO) staging for carcinoma of the ovary [8]. The patient was again discussed on the tumor board. Surgery was not considered because of the risk of bleeding since the tumor was highly vascular and fragile. She received systemic neoadjuvant chemotherapy consisting of weekly paclitaxel (80 mg/m2) plus carboplatin (AUC2). She was further examined for surgery after three courses of neoadjuvant chemotherapy. After that, the patient underwent a total abdominal hysterectomy, bilateral salpingo-oophorectomy, infracolic omentectomy, bilateral pelvic lymph node dissection, paraaortic lymph node sampling, peritoneal cytology sampling, and appendectomy. The final pathology consisted of stage IIIC high-grade tubo-ovarian serous carcinoma. Postoperatively, she was recommended cisplatin/paclitaxel chemotherapy. It has been eight months since the patient's first diagnosis, and in the last imaging performed two months ago, no metastasis was detected.

## Discussion

Metastasis of ovarian cancer to the female genital tract is a rare entity. Half of the secondary tumors to the female genital tract are of GI origin. While genitourinary, breast and urothelial neoplasms can metastasize to this region [9]. Mazur MT et al. reviewed 325 metastatic cancers from 269 patients treated at Barnes Hospital between 1950 and 1981 and reported that 149 out of 325 cases were with extragenital primaries in which the majority with adenocarcinomas of the GI tract [10]. One hundred and seventy-six of these cases were with genital metastases. Among these genital cases, seven of them had cervical metastasis, and all of them had primaries of ovarian carcinoma.

Cervical metastasis of ovarian cancer is extremely rare. Lemoine NR and Hall PA reported that only 12 of the ovarian cancer cases developed cervical metastasis in London Hospital in the 65-year study period [11]. Guidozzi et al. [12] studied 148 cases of stage 3-4 ovarian cancer. Out of 148 cases, only seven patients had a metastatic cervical mass at the time of diagnosis. Four were direct extensions of the primary lesion, while three were true metastases. Malignant ascites, retroperitoneal lymph node involvement, and peritoneal carcinomatosis were observed in all patients with cervical metastasis.

Our case had specific features. She had a huge cervical mass mimicking a true cervical tumor that invaded the whole vagina. Despite the giant tumor, the cervical stromal ring was intact in T2-weighted images, which was unusual for cervical carcinoma. There was no parametrial invasion or any suspicious lymph nodes. However, both MRI and PET imaging detected pelvic peritoneal implant and bilateral adnexal masses. The FDG uptake was much higher in the cervical tumor than in the adnexal masses. Therefore, it was easy to consider the patient as having advanced cervical cancer. It was decided to take another biopsy from the predominant mass located in the cervix and vagina since the second opinion by an experienced gynecologic pathologist did not confirm the diagnosis of SCC. It was found that high-grade tubo-ovarian cancer did metastasize to the cervix.

Zannoni GF et al. examined 144 cases that metastasized to the cervix and observed that 23 cases originated from the ovary. The pathological diagnoses of 20 out of 23 cases were high-grade serous carcinoma confirmed by diffuse staining with WT1 [13]. The patients in the study by Zannoni et al., similar to the others in the literature, were all with widespread peritoneal seeding. Our case had also shown diffuse staining with WT1. However, different from the series in the literature, our patient did not have widespread peritoneal seeding though she had an implant in the pelvic peritoneum. In addition, there was much higher FDG uptake in cervical mass than the primary ovarian masses in our case. We thought that this significant FDG uptake in the cervical metastatic mass showed the aggressive behavior of the tumor. We then decided to treat the patient with systemic chemotherapy first after a course of palliative external pelvic RT in order to stop the vaginal bleeding.

Although cervical metastasis is rare, such lesions can be insidious and mimic the primary disease, which is a difficult differential diagnosis for both the clinician and the pathologist. However, it is crucial to determine the true origin of the prognosis of the disease and the treatment approach. In our case, ovarian cancer presented with cervical metastasis and was approached as cervical cancer at first glance. However, our case is also unusual for the natural presentation and clinical progression of cervical cancer. In such cases, the presence of a single cervical mass in primary cervical malignancies, presence of concomitant in situ foci, and presence of koilocytosis in histopathology are the key elements recommended in the differentiation of primary cervical cancer and metastasis. In those with secondary cervical malignancies, multiple masses, cervical tumor in the outer layer of the cervix and spared surface epithelium, signet-ring cells, an Indian file pattern, glandular entrapment, and widespread vascular and lymphatic involvement are key elements to differentiate it from the primary [13, 14].

## Conclusions

Although metastasis of ovarian cancer to the cervix is ​​rarely seen, it should be kept in mind that a tumor in the female genital tract that deviates from the usual course of the disease may be a secondary lesion rather than a primary tumor. This distinction will be crucial in determining the patient's treatment. Our case distinguishes from its counterparts by the absence of advanced-stage disease with malignant ascites, retroperitoneal lymph node involvement, and peritoneal carcinomatosis. A multidisciplinary approach is essential in properly evaluating this group of patients.
